# Establishment and comparison of air-liquid interface culture systems for primary and immortalized swine tracheal epithelial cells

**DOI:** 10.1186/s12860-018-0162-3

**Published:** 2018-06-28

**Authors:** Haiyan Wang, Lina He, Beibei Liu, Yanyan Feng, Hao Zhou, Zhenzhen Zhang, Yuzi Wu, Jia Wang, Yuan Gan, Ting Yuan, Meng Wu, Xing Xie, Zhixin Feng

**Affiliations:** 1 Institute of Veterinary Medicine, Jiangsu Academy of Agricultural Sciences, Key Laboratory of Veterinary Biological Engineering and Technology, Ministry of Agriculture, 50 Zhongling Street, Nanjing, 210014 China; 2 College of Veterinary Medicine, Nanjing Agricultural University, Nanjing, 210095 China

**Keywords:** Air-liquid interface, Immortalized swine tracheal epithelial cell line, Primary swine tracheal epithelial cells, Differentiation, comparison

## Abstract

**Background:**

Air-liquid interface (Ali) systems allow the establishment of a culture environment more representative of that in vivo than other culture systems. They are useful for performing mechanistic studies of respiratory epithelial cells as drug permeation barriers and can be used to study the interactions between hosts and respiratory pathogens. However, there have been few studies concerning Ali cultures of primary swine tracheal epithelial cells (STECs) and an immortalized STEC line, and the differences between these two systems remain poorly defined.

**Results:**

In this study, we established Ali culture systems for primary STECs and for immortalized STEC line, and we systematically compared the differentiation capacities and immunological functions of these systems for the first time. Under Ali culture conditions, immortalized STEC line and primary STECs could survive for at least forty days, formed tight junctions and differentiated into stratified cells. They both possessed complete abilities to produce mucin and inflammatory cytokines and develop cilia. However, in contrast to primary STECs, which had a heterogeneous morphology, Ali-cultured immortalized STEC line appeared to be a homogenous population. The formation of tight junctions in Ali-cultured primary STECs was superior to that in immortalized STEC line. In addition, cilia in Ali-cultured immortalized STEC line were more pronounced, but their duration of expression was shorter than in primary STECs.

**Conclusions:**

Ali-cultured primary STECs and immortalized STEC line systems possessing complete abilities to undergo ciliary differentiation and inflammatory cytokine production were established for the first time in this study, and several differences in morphology and the formation of tight junctions and cilia were observed between these two systems. These two systems will be important tools for drug screening studies, as well as for detailed analyses of the interactions between hosts and respiratory pathogens.

## Background

The first physical barrier in the airway that an inhaled microorganism encounters is the respiratory epithelium [1]. Mucus on the surface of the respiratory epithelium forms a protective coating that captures inhaled microorganisms, facilitating their clearance by mucociliary transport [2]. The respiratory epithelium also plays an important role in regulating innate and acquired immunity through the production of a wide range of cytokines [3, 4]. Primary respiratory epithelial cells consist of multiple cell types, such as goblet cells, basal cells, and ciliated epithelial cells; goblet cells produce mucus, and cilia are developed by ciliated epithelial cells [5, 6].

In an attempt to better mimic the native conditions of the airway epithelium in vivo, air-liquid interface (Ali) culture systems using primary airway epithelial cells or immortalized cell lines of humans, mice, swine and sheep have been developed [1, 7–10]. Ali culture systems offer many advantages over submerged cell culture systems. For example, they can enable the in vitro reconstitution of a pseudo-stratified epithelium that possesses many features comparable to those observed in vivo, including the production of cilia and visible mucus [8–10]. However, certain challenges currently hinder the wide application of Ali culture systems for primary airway epithelial cells or immortalized cell lines [3, 9, 10], especially for the airway epithelial cells of swine. Only a handful of studies concerning this methodology have been reported [11, 12]. The abilities to undergo ciliary differentiation and produce mucus in these systems are immature or lost, and the differences in phenotype and function between Ali-cultured primary swine tracheal epithelial cells (STECs) and immortalized STEC line remain unclear. To perform appropriate drug transport mechanism studies or studies of the interactions between pathogens and the host, it is necessary to clarify the regulatory mechanisms of differentiation and immunological function of Ali cultured cells in vitro. In this study, Ali culture systems for primary STECs and for immortalized STEC line were established, and a systematic comparison of the morphology, differentiation capacity and immunological function of these two systems was performed for the first time. Ali-cultured immortalized STEC line and primary STECs systems will be important tools for the study of host-pathogen interactions and drug screening.

## Methods

### Air–liquid interface (Ali) culture of an immortalized swine trachea epithelial cell (STEC) line

Immortalized STEC line (Cat.No. x1204502) used in this study were purchased from Shanghai Fu Sheng Industrial Co.,Ltd. in China. Immortalized STEC line (between 10 and 20 passages) were cultured in RPMI 1640 medium (Invitrogen, Carlsbad, CA, USA) supplemented with 10% fetal bovine serum (FBS, Invitrogen, Carlsbad, CA, USA), 20 IU/ml of penicillin and 20 mg/ml of streptomycin (Invitrogen, Carlsbad, CA, USA). Cells were cultured at 37 °C in 5% CO2 in a humidified atmosphere. Sub-passages were made when cells reached 100% confluence. After trypsinization, collected cells were seeded onto type IV collagen (Sigma)-coated transwell permeable supports (6.5 mm or 24 mm, 0.4 μm polyester membrane, Corning, NY, USA) at a density of 10^5^ cells/ml. A total of 0.8 ml of fresh medium was added to the lower reservoir, and 0.25 ml of cell suspension was added to the upper reservoir. After 48 h of incubation at 37 °C in 5% CO2 in a humidified atmosphere, when cells were completely confluent, the Ali was formed by removing the apical medium, and the cells were fed with medium only from the basal compartment. The number of days of development was designated relative to the initiation of Ali culturing, which corresponded to day 1 (D1). Cultures were maintained under Ali conditions for at least twenty-one days.

### Isolation and Ali culture of primary swine trachea epithelial cells (STECs)

Based on serologic testing (enzyme-linked immunosorbent assay (ELISA)), three pigs (2 or 3-month-old, female, Large White) free from porcine reproductive and respiratory syndrome virus (PRRSV), swine influenza virus (SIV) and *Mycoplasma hyopneumoniae* (Mhp) were used in this study. These pigs were from a farm in Nanjing, China. Primary STECs were isolated as described previously with slight changes [11]. Tracheas from healthy pigs were dissected and cut into 1 × 2 cm portions. Then, the tracheas were washed in chilled D-Hank’s solution three times and placed in a pronase/DNAse solution containing minimum essential medium (MEM, Invitrogen, Carlsbad, CA, USA), 1 mg/ml pronase (Sigma, St.Louis, MO, USA) and 100 μg/ml DNAse (Sigma) for 24–48 h at 2–8 °C. Enzymatic dissociation was terminated by adding FBS at a final concentration of 10%. Cells were then harvested by centrifugation at 500×*g* for 10 min, resuspended in Dulbecco’s modified Eagle’s medium (DMEM, Invitrogen, Carlsbad, CA, USA) with 10% FBS, and then incubating on tissue culture plates for 2 h (Corning, NY, USA). The fibroblasts were removed via their differential adherence to plastic. Epithelial (non-adherent) cells were collected by centrifugation and resuspended in bronchial epithelial growth media (BEGM, Lonza, Walkersville, MD, USA). Then, the cells were seeded into collagen-coated transwell permeable supports (6.5 mm or 24 mm, 0.4 μm polyester membrane, Corning, NY, USA) at 3–5 × 10^6^ cells/ml. A total of 0.6 ml of BEGM was added to the lower reservoir, and 0.2 ml of cell suspension was added to the upper reservoir. After 24 h, when cells were completely confluent, the Ali was created by removing the apical medium, and the medium was changed to a differentiation medium that consisted of BEGM, 2% UltroserG serum substitute (USG, Pall, NY, USA), and retinoic acid (15 ng/ml, Sigma). The culture was maintained under Ali conditions for at least 21 days.

### Measurement of trans-epithelial electrical resistance (TEER)

Trans-epithelial electrical resistance (TEER) was measured using a Millicell ERS volt-ohm meter (Millipore, Molsheim, France). On day 1 and every 2 days through day 21 under Ali conditions, 150 μl of medium was added apically into the insert, and measurements were performed. Before measuring the TEER of each culture, an empty culture insert was used as a blank, and the measured value was subtracted from each subsequent sample value. After the measurement, the apical medium was discarded to restore Ali conditions. Data are presented as resistance values (Ωcm^2^) and given as the mean +/− standard deviation (SD) of three experiments, each done in triplicate (*n* = 9).

### Indirect immunofluorescence assays (IFAs)

Indirect immunofluorescence assays (IFAs) were performed as previously described [13]. Cell membranes were permeabilized by 0.2% TritonX-100 in phosphate buffered saline (PBS) for 5 min and blocked in 1% bovine serum albumin (BSA) for 30 min before incubation with primary and secondary antibodies. Primary antibodies against mucin 5B (MUC5B) (1:100 dilution; Abcam, Cambridge, UK) or the *zona occludens-1* protein (ZO-1, 1:100 dilution; Abcam) in 1% BSA were incubated with cells overnight at 2–8 °C. Then, cells were washed and incubated for 1 h with Alexa Fluor 488-conjugated goat anti-mouse IgG (1:400, Beyotime Biotech, Nantong, China) or Alexa Fluor 555-conjugated goat anti-rabbit IgG (1:400, Beyotime Biotech). The cells were further stained with 2,4-diamidino-2-phenylindole (DAPI; Beyotime Biotech) for 5 min at room temperature and washed with PBS. Cells were then imaged using an LSM 710 laser scanning confocal microscope (Zeiss, Germany).

### Scanning electron microscopy (SEM) analysis

Filter membranes with immortalized STEC line or primary STECs were washed in PBS and fixed in 2.5% glutaraldehyde in 0.1 M phosphate buffer (pH 7.4). Then, specimens were dehydrated in a graded series of acetone and dried in a hexamethyldisilazane (HMDS) solution (Sigma-Aldrich). Dried specimens were coated with a thin layer of platinum with an ion beam coater using a Precision Etching and Coating System (PECS; Gatan France, Evry, France) and observed with a Zeiss Ultra+Field Emission Gun Scanning Electron Microscope (FEGSEM; Carl Zeiss S.A.S, Le Pecq, France).

### Histological examination

After cultures were grown for two weeks under Ali conditions, the membranes were removed from the transwell, cultures were fixed with 4% polyoxymethylene (Beyotime Biotech), and samples were embedded in wax, cut in transverse sections, and stained with hematoxylin and eosin (H&E). Ciliogenesis was assessed by counting the number of ciliated cells (cells with cilia attached to the apical surface) per randomly chosen microscope field (400 × magnification) as previously described [11]. Data are given as the mean +/− SD of three microscope fields per culture and three independent cultures (*n* = 9).

### Cytokine quantification

The Ali-cultured immortalized STEC line and primary STECs were exposed to Toll-like receptor (TLR) agonists (Sigma), including 0.1 μg/ml FSL-1 (a synthetic diacylated lipoprotein; TLR2/6 ligand), 100 ng/ml LPS (purified lipopolysaccharide from *Salmonella enterica* subsp. *enterica* serovar Minnesota; TLR4 ligand), and 25 μg/ml poly I:C (synthetic analog of double-stranded RNA; TLR3 ligand). Cellular supernatants were collected from each well 24 h post stimulation and stored at below − 70 °C until analysis. Cytokine analysis was carried out with tumor necrosis factor alpha (TNFα) and interleukin-1 beta (IL-1β) ELISA kits (Abcam) according to the manufacturer’s instructions. Data are presented as the ratios of treated vs control cells for each stimulant in immortalized vs primary cells, and given as the mean +/− SD of three experiments, each done in triplicate (*n* = 9).

### Statistics

The results were tested for normality by Shapiro-Wilk test using GraphPad Prism (GraphPad, San Diego, CA), *P > 0.05* indicated the distribution of the data was normal. Then they were analyzed for significance by t-tests (GraphPad), *P < 0.05* indicated significant differences between the 2 groups.

## Results

### The development of immortalized STEC line and primary STECs cultured under Ali conditions

**Fig. 1:**
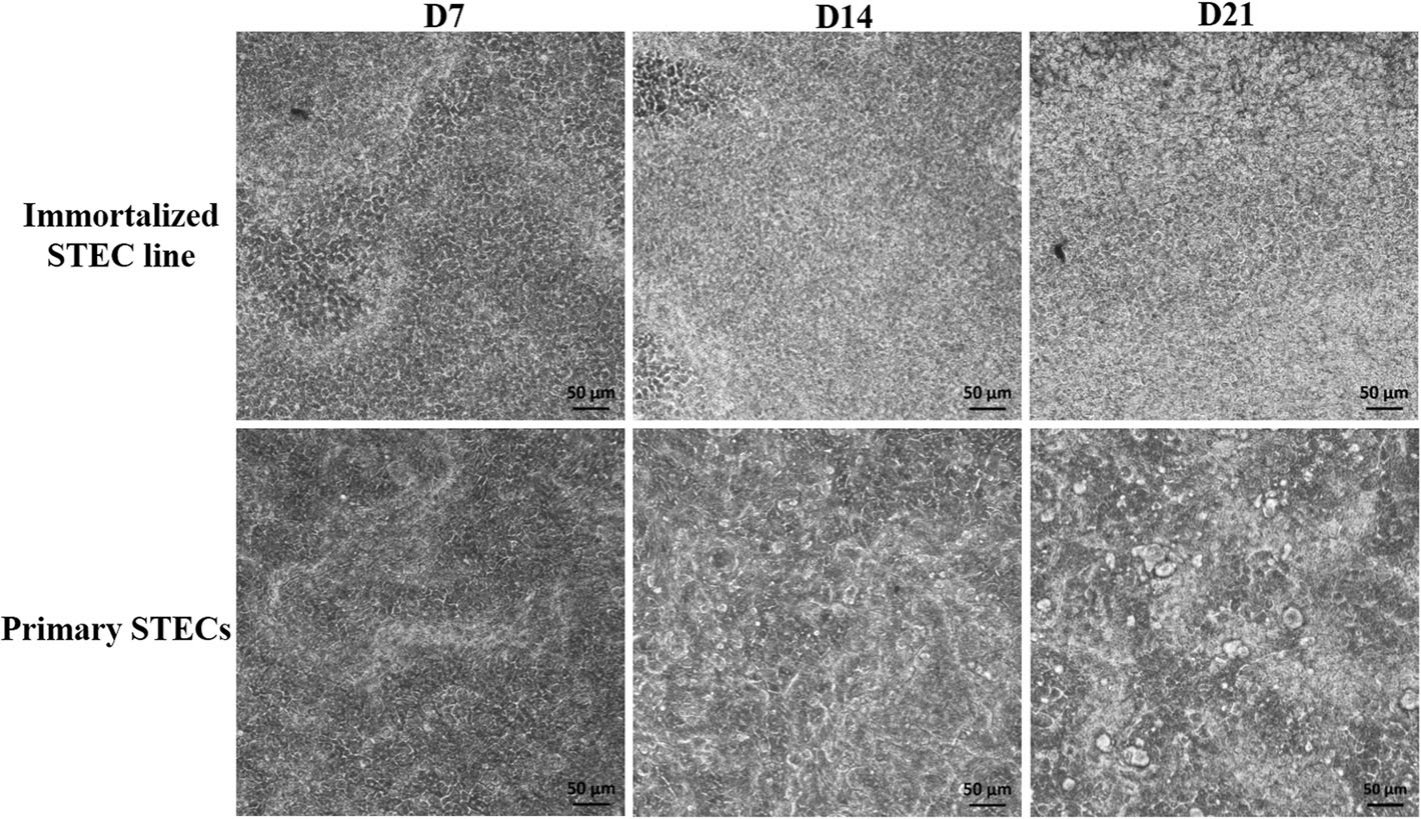
Development of immortalized STEC line and primary STECs cultured under Ali conditions over 21 days. The immortalized STEC line and primary STECs were immersed in media, and they grew into a confluent monolayer within 2 days. At this time, the apical medium was then discarded to create Ali conditions. Cellular morphological changes at different time points (D7, D14, and D21) were observed by conventional light microscopy

The immortalized STEC line and primary STECs were immersed in media, and they grew into a confluent monolayer within 2 days. At this time, apical medium was then discarded to create Ali conditions. The immortalized STEC line and primary STECs could survive for at least 40 days under Ali conditions. Cellular morphological changes at different culture times were observed initially by conventional light microscopy. From D7 to D21, the Ali-cultured immortalized STEC line appeared to be a homogenous population of epithelial cells. In contrast to the immortalized STEC line, the morphology of primary STECs was heterogeneous; with increasing culture time, morphological diversity increased. In addition, both the immortalized STEC line and primary STECs displayed lighter areas as early as D7, likely indicative of stratified cells, and these lighter areas corresponded to more dense regions (Fig. 1).

### Trans-epithelial electrical resistance (TEER) and ZO-1 protein expression assessment

**Fig. 2:**
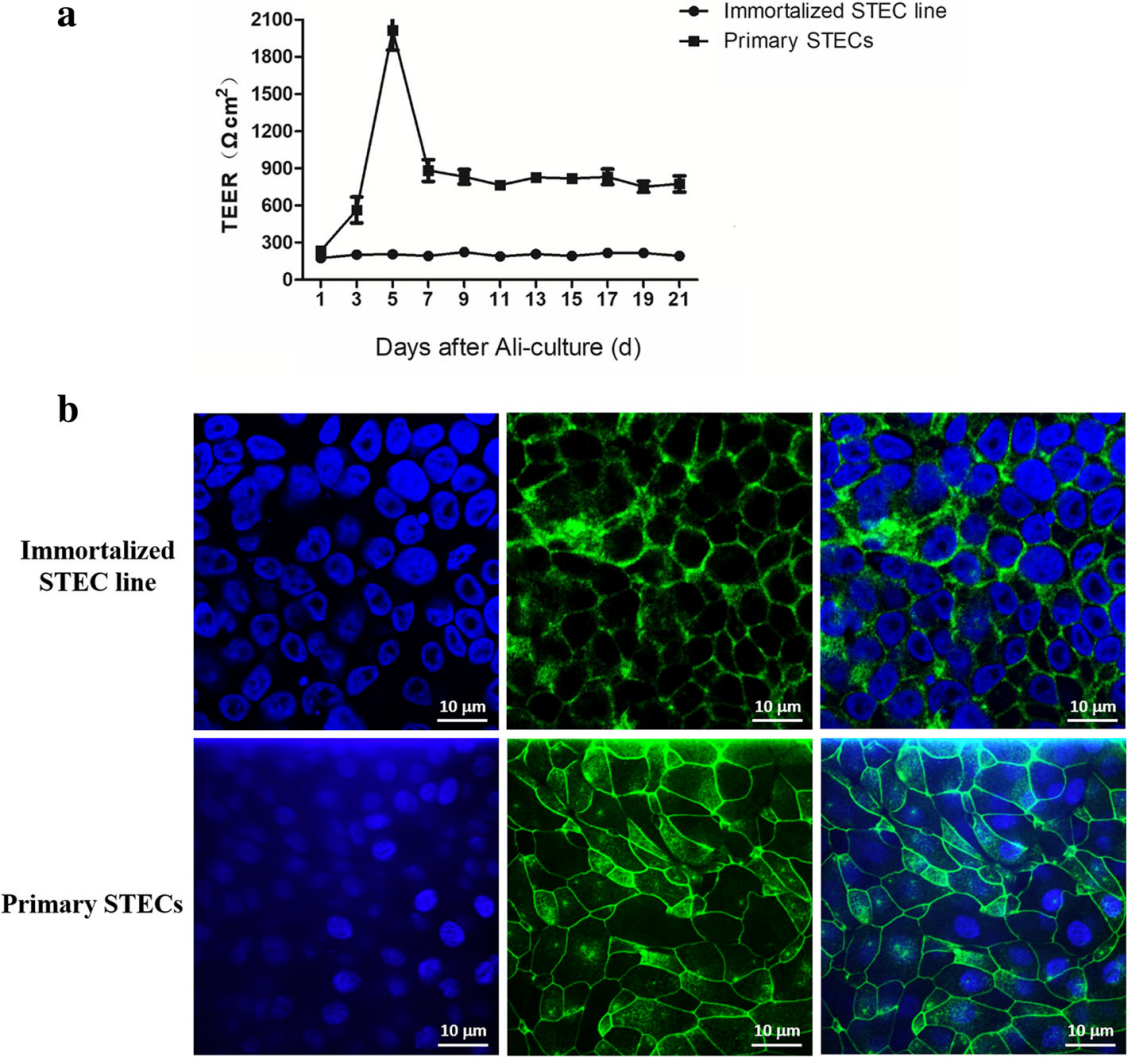
Assessment of tight junctions in immortalized STEC line and primary STECs cultured under Ali conditions. Immortalized STEC line and primary STECs were cultured under Ali conditions for 21 days. TEER was measured on D1 and every 2 days thereafter through D21 under Ali conditions. Data are presented as resistance values (Ωcm^2^)and given as the mean +/− SD of three independent experiments, each done in triplicate (*n* = 9) (**a**). At D7, the cells were fixed with 4% paraformaldehyde and stained with an anti-ZO-1 antibody, followed by incubation with a Alexa Fluor 488-conjugated goat anti-mouse secondary antibody. The cells were then counterstained with DAPI and imaged using laser scanning confocal microscopy (**b**)

Trans-epithelial electrical resistance (TEER) data for immortalized STEC line throughout cell culture development displayed remarkably stable values (approximately 200 Ωcm^2^). In contrast to immortalized STEC line, the Ali-cultured primary STECs reached a maximum TEER (2000 Ωcm^2^) as early as D5 and then gradually decreased to 800 Ωcm^2^; this level was maintained until D21 (Fig. 2a). ZO-1 (a major component of the tight junction) levels in Ali-cultured primary STECs and immortalized STEC line were detected by using an anti-ZO-1 antibody. ZO-1 proteins appeared to reach the cell membrane/cell-cell junctions in both Ali-cultured primary STECs and immortalized STEC line, as expected. However, tight junctions were more heavily and distinctly stained in primary STECs than in immortalized STEC line, suggesting that the formation of tight junctions was superior in the former cells, which is consistent with the TEER findings (Fig. 2b).

### Scanning electron microscopy (SEM) analysis

**Fig. 3:**
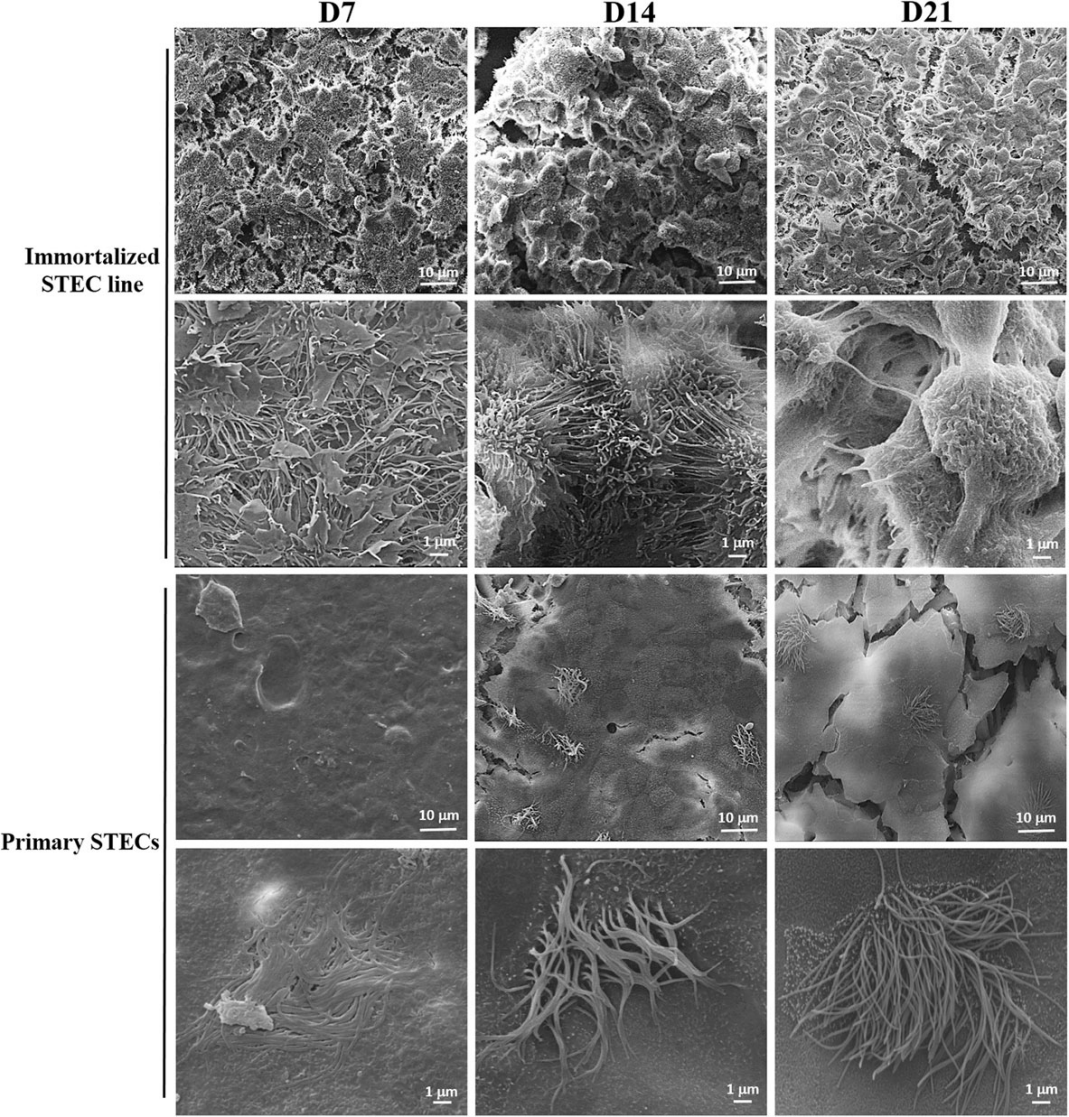
Scanning electron microscopy (SEM) analysis of immortalized STEC line and primary STECs cultured under Ali conditions. Immortalized STEC line and primary STECs were cultured under Ali conditions for 21 days, and cilia differentiation on the cells was evaluated by SEM at D7, D14 and D21

SEM analysis revealed flattened, possibly being immature cilia to be present at D7. They increased in length and density to D14, at which time mature cilia were presented on both primary STECs and immortalized STEC line. The cilia on immortalized STEC line were more pronounced than that on primary STECs at D7 and D14. However, at D21, cilia were degraded on immortalized STEC line, whereas they remained visible on primary STECs (Fig. 3).

### Histological examination

**Fig. 4:**
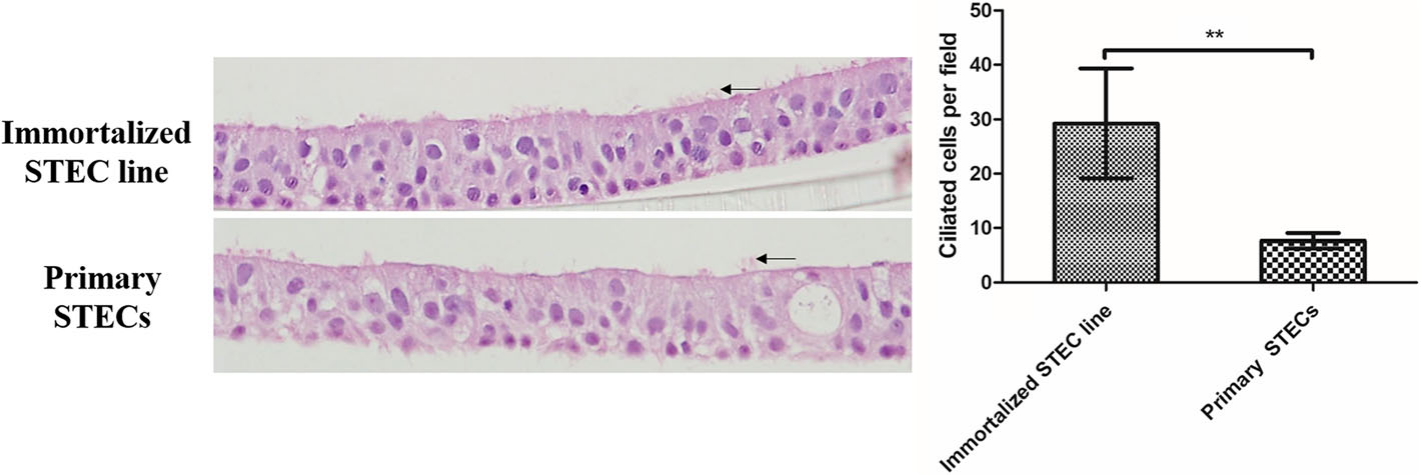
Histological examination of immortalized STEC line and primary STECs cultured under Ali conditions. At D14, the cultures were fixed with 4% polyoxymethylene, then followed by histological sectioning and H&E staining. Ciliogenesis was assessed by counting the number of ciliated cells per randomly chosen microscope field (400 × magnification). Data are given as the mean +/− SD of three microscope fields per culture and three independent cultures (*n* = 9). (*P < 0.05, **P < 0.01)

Histological examination was used to quantify the ciliogenesis on primary STECs and immortalized STEC line at D14. As shown in Fig. 4, primary STECs and immortalized STEC line both formed stratified cells, and columnar ciliated cells were found on their surface (Black arrow). Ciliogenesis was assessed by counting the number of ciliated cells in randomly chosen microscope field for each of three independent cultures, and the mean number of ciliated cells in immortalized STEC line was significantly more than in primary STECs (*P < 0.01*). In conclusion, cilia in Ali-cultured immortalized STEC line were more pronounced, but their duration of expression was shorter than in primary STECs.

### Mucin expression analysis

**Fig. 5:**
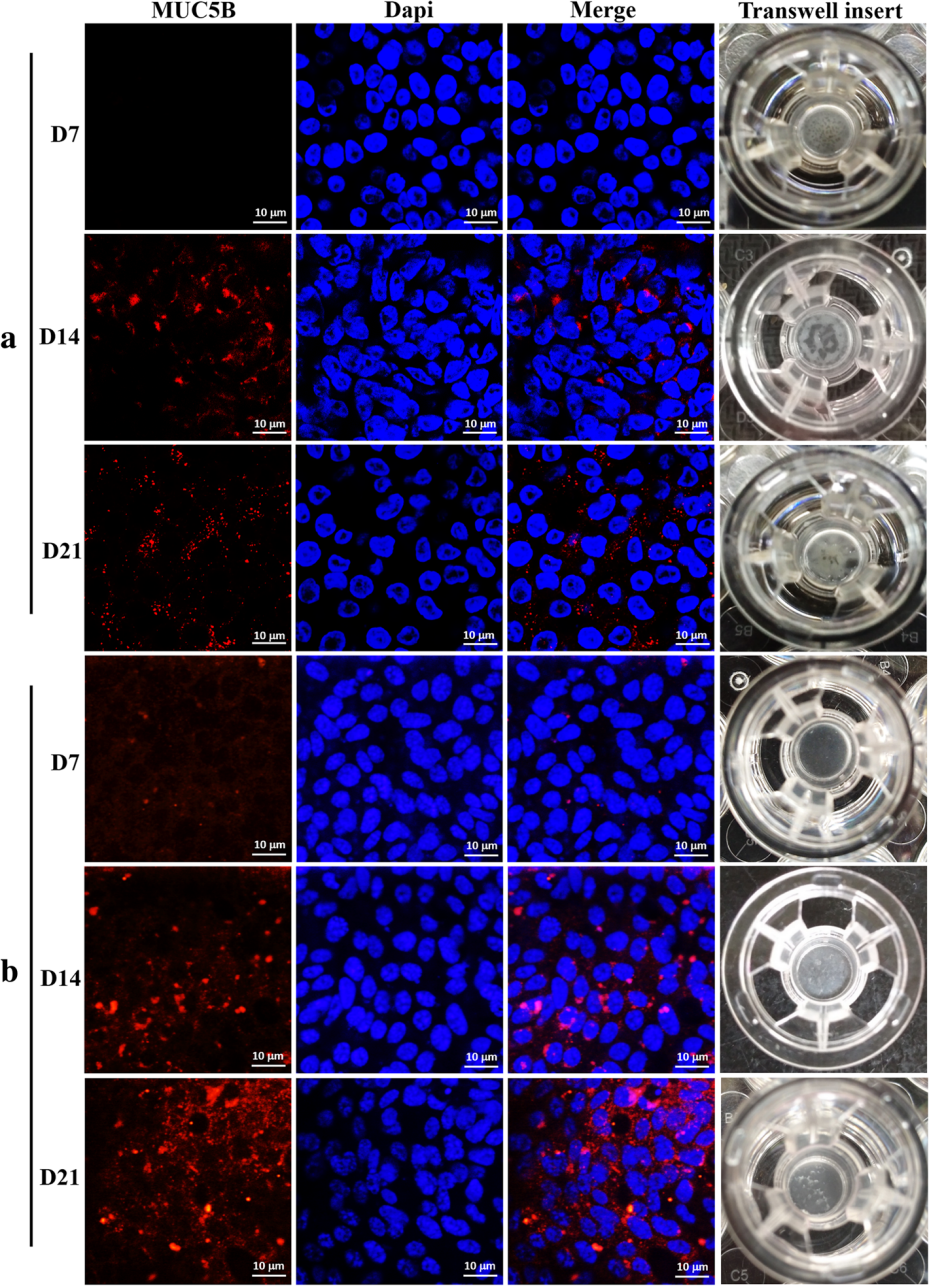
Mucus secretion by immortalized STEC line and primary STECs cultured under Ali conditions. Immortalized STEC line and primary STECs were cultured under Ali conditions for 21 days. At D7, D14, and D21, the cells were fixed with 4% paraformaldehyde and stained with an anti-MUC5B antibody, followed by incubation with a Alexa Fluor 555-conjugated goat anti-rabbit secondary antibody. The cells were then counterstained with DAPI and imaged using laser scanning confocal microscopy (**a**, **b**)

Mucins are continuously secreted by intraepithelial goblet cells and are composed of large glycoproteins that crosslink to form a structural barrier [14]. By D7, there was no visible secreted material on the airway surface of immortalized STEC line cultures. Starting at D7, secretion increased over time, becoming markedly visible at D14 and widely distributed in the majority of immortalized STEC line at D21 (Fig. 5a). In contrast to immortalized STEC line, secreted material on the airway surface of primary STECs cultures was visible until D21 (Fig. 5b). MUC5AC and MUC5B are the major mucins in human airways [2]. To determine the differentiation ability of immortalized STEC line and primary STECs, the expression of MUC5B was analyzed by immunofluorescence staining with a MUC5B-targeting antibody. The results indicated that MUC5B was present in immortalized STEC line starting at D14, and these cells exhibited robust production at D21. It was mainly distributed around the nucleus, with an aggregated distribution at D14 and a scattered distribution at D21, which was similar to the findings in primary STECs. However, the complete composition of the secreted material on the airway surface of these cultures requires further research.

### Induction of inflammatory cytokines in Ali-cultured immortalized STEC line and primary STECs in response to TLR agonists

**Fig. 6:**
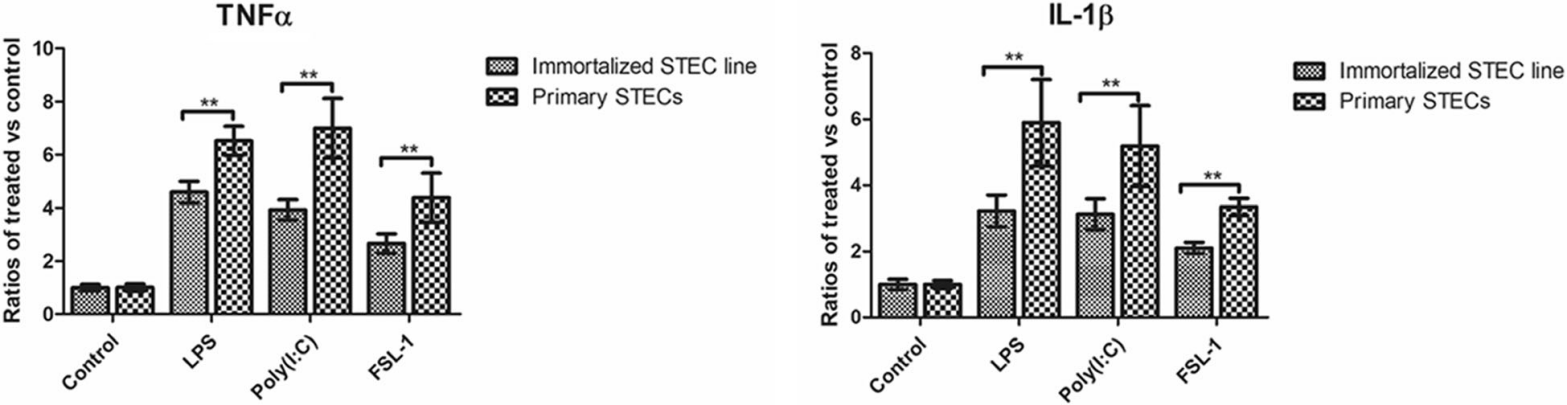
Induction of pro-inflammatory cytokines in Ali-cultured immortalized STEC line and primary STECs in response to Toll-like receptor (TLR) agonist stimulation. Immortalized STEC line and primary STECs were cultured under Ali conditions. At D14, the cells were treated with TLR agonists for 24 h. The cellular supernatants were collected, and cytokine analysis was performed with TNFα and IL-1β ELISA kits. Data are presented as the ratios of cytokine expression by treated cells relative to expression by control cells in immortalized or primary cells, and given as the mean +/− SD of three experiments, each done in triplicate (*n* = 9). (*P < 0.05, **P < 0.01)

The airway epithelium has recently been recognized as participating in the inflammatory response by producing a number of pro- and anti-inflammatory factors [8, 14]. TLRs play a major role in eliciting inflammatory responses [15]. To determine whether the Ali-cultured immortalized STEC line and primary STECs possessed functioning and responsive TLRs, a panel of TLR agonists LPS (TLR4, bacterial product), FSL-1 (TLR2/6, mycoplasmal product), and polyI:C (TLR3, viral product) were used to treat Ali-cultured cells at previously published concentrations [15]. After 24 h of exposure, Ali-cultured immortalized STEC line responded with inflammatory cytokine (TNFα, IL-1β) secretion, which was also observed in primary STECs. The ratios of treated vs control cells for each stimulant in primary STECs were significantly higher than that in immortalized STEC line (*P < 0.01*) (Fig. 6).

## Discussion

Compared to the use of submerged epithelia, Ali systems allow the creation of a study environment more representative of that in vivo. These systems are useful for performing mechanistic studies on the function of epithelial cells as drug permeation barriers, and they can be used to study interactions between hosts and pathogens [1, 10]. Regarding respiratory cell models, there are primary cells and immortalized cell lines available from different locations in the respiratory tract, including tracheal, bronchial and alveolar epithelial cells [10, 16, 17]. As the first line of lung defense, the airway epithelium provides a mucosal barrier to prevent infection via ciliary motion, mucus production, and tight-junction formation, and the epithelium produces cytokines that are important mediators for regulating innate and acquired immunity [2–4]. In this study, we established two Ali culture systems; one for primary STECs and another for immortalized STEC line. The differentiation capacity and immunological function of the two systems, as well as their morphology, were compared and analyzed for the first time.

When cells are used for drug or vaccine transport studies in an insert system, cell permeability is a key factor [18]. TEER measurements are typically used for the evaluation of the cell permeability in transwell inserts [9, 17, 18]. Previous studies have shown that cell layers in insert systems under different culture conditions may show differences in TEER [9, 17, 19]. TEER analysis of Ali-cultured newborn pig trachea (NPTr) cell lines (passage #30–50) showed values of approximately 150 Ωcm^2^ in the first 18 days [9]. Similar to those of NPTr cells, TEER values of immortalized STEC line in this study (passage #10–20) were quite stable (approximately 200 Ωcm^2^) throughout the period of cell culture development. In contrast to the immortalized cell line, the Ali-cultured primary STECs showed a higher but more variable TEER, which increased until D5 and then quickly decreased from approximately 2000 Ωcm^2^ to 800 Ωcm^2^. This interesting result has also been found in several previous reports [12, 18]. According to previous reports, variation in the TEER of Ali-cultured primary cells also occurs at different passages in vitro [18, 20]. Primary cultured normal human bronchial epithelial (NHBE) cells showed an increase in their maximum TEER to nearly 1000 Ωcm^2^ in the 2nd passage, whereas it decreased to approximately 600 Ωcm^2^ in the 3rd passage and was < 150 Ωcm^2^ in the 4th passage. TEER may decrease with the continued passaging of cells, which may contribute to a reduced but stable TEER in immortalized cells compared to that in primary cells. The nature of serums used for the differentiation of the two cells may also bring differences in TEER. One of the serums was bovine serum, and the other was UltroserG serum substitute (USG). In addition to USG, there were some supplements, such as growth factors, hydrocortisone contained in BEGM medium, which may be more conducive to the development of TEER. In addition, TEER can also be variable between cells from different donors. Cells from asthmatic subjects showed decreased TEER compared to the TEER of normal cultures of cells from healthy subjects [20]. Therefore, the health status of donors can affect the development of TEER. The pigs used in this study were healthy and free from PRRSV, SIV and Mhp infection, which may have contributed to the development of a high TEER.

The protein ZO-1 is reportedly one of the most important cytosolic proteins for tight-junction formation, which is related to cell layer permeability. The strong positive ZO-1 staining in primary STECs is consistent with the increase in TEER and shows that the ZO-1 protein is necessary for tight-junction formation among primary STECs. For Ali-cultured immortalized cells, ZO-1 staining was much weaker than in primary cells. Similar results have previously been reported in a NPTr cell line and a human airway epithelial cell line, Calu-3 [9, 10], which suggests that in addition to ZO-1, other ZO proteins or cytosolic proteins may contribute to tight-junction formation in immortalized cells.

Colonization of the airways requires evasion of mucociliary defenses that effectively capture and remove inhaled substances, limiting their access to the epithelium [21]. In vitro, when STECs are maintained in submerged conditions, the differentiation of epithelial cells into ciliated cells is strongly suppressed [9]. Ali culture systems increase the oxygen supply to a level that better meets the requirements of airway epithelial cells and can promote differentiated phenotypes to an extent similar to that observed in vivo [8–10]. However, Delgado-Ortega reported that microvilli but not cilia differentiation was observed in Ali-cultured NPTr cells [9, 10]. In this study, SEM results revealed cilia differentiation in both immortalized STEC line and primary STECs when cultured in an Ali system. NPTr cell lines were established from a 2-day-old piglet, whereas the immortalized STEC line in our study were from a 2-month-old piglet. We speculate that the immature differentiated phenotype of STECs in the 2-day-old piglet may have contributed to the cilia loss observed in Ali-cultured NPTr cells. The same reasoning is also relevant for infection by Mhp, a pathogen that colonizes ciliated epithelial respiratory cells and induces *Mycoplasmal pneumonia* in swine [22, 23]. Mhp infection in piglets usually begins during the late stages of lactation and becomes increasingly severe during the finishing period, which may be due to the lack of cilia differentiation in newborn piglets. Therefore, evaluation of cilia differentiation in Ali-cultured epithelial cells is important, especially for studies of pathogens targeting ciliated cells. In this study, cilia on primary STECs were observed as early as D7 and continued to develop from D14 to D21. However, cilia differentiation in immortalized STEC line increased and was more pronounced from D7 to D14. The immortalized STEC line formed a homogenous population of epithelial cells, but the primary STECs consisted of a mixture of cell types. The higher purity and number of ciliated cells may be responsible for the earlier and higher production of cilia in immortalized STEC line.

Airway mucus forms a protective coating that entraps foreign particles and microbes, facilitating their clearance by mucociliary transport, and a deficient mucus barrier leaves the lungs vulnerable to injury [2]. MUC5AC and MUC5B are the major mucins in human airways [24]. In Ali-cultured NHBE cells, there are basal levels of MUC5AC and MUC5B expression [2, 21, 25]. The production of mucus has also been observed in NPTr cells [9]. Mucin secretion was observed from D15 to D17 in Ali-cultured NHBE cells, and these cells exhibited robust production from D43 to D45 [1, 21]. Mucus secretion in primary STECs was similar to that in NHBE cells, while mucus secretion occurred slightly earlier in immortalized STEC line than in NHBE cells. A MUC5B-specific monoclonal antibody was used to clarify mucus secretion characteristics in unstimulated Ali-cultured cells. However, no MUC5B-specific antibody staining was observed at D7. MUC5B was obviously expressed in Ali-cultured immortalized STEC line and in primary STECs at D14, and these cells exhibited robust MUC5B production at D21, with similar secretion profiles. It is speculated that the mucin composition of primary STECs and immortalized STEC line may be different. In addition, excessive mucus secretion may be a mechanism leading to infection-induced exacerbation of airway diseases [25]. Some airway pathogens, such as *Mycoplasma pneumoniae* could induce the expression of MUC5AC and MUC5B [2, 25]. Therefore, evaluations of MUC5B staining in cells stimulated by specific porcine airway pathogens will be the subject of future studies.

The airway epithelium has recently been recognized to participate in the inflammatory response by producing a number of pro- and anti-inflammatory factors [14]. *Mycoplasma ovipneumoniae* induces inflammatory responses in Ali-cultured sheep airway epithelial cells, indicating that the Ali culture system is a reliable model for investigating host-pathogen interactions [8]. In this study, the inflammatory cytokines TNFα and IL-1β were expressed in Ali-cultured immortalized STEC line and primary STECs in response to TLR agonist stimulation. The ratios of treated vs control cells for each stimulant in primary STECs were significantly higher than that in immortalized STEC line. Mhp is an extracellular bacterium that colonizes the respiratory epithelium of pigs and induces the production of a number of inflammatory cytokines in infected pigs [26]. Therefore, Ali-cultured STEC line and primary STECs may also be reliable models for investigating Mhp-host interactions.

Primary cells are more physiologically relevant to in vivo organs, but during the isolation of primary cells, contamination with micro-organisms and fibroblasts is common [9, 20]. Many factors, including sample type, underlying diseases, sample handling, cell isolation and culture techniques and the medium used may affect the fate of primary airway epithelial cultures [3]. In addition, there is a high degree of variability between donors and cells from different passages [20]. Compared with primary cells, immortalized cell lines have several advantages, including a longer life span, lower cost, and lower variability between passages and experiments. However, the number of passages is critical, and cells will undergo a dedifferentiation process and lose their phenotype at later stages [3, 9]. Several airway epithelial cell lines cultured under Ali conditions do not possess complete differentiation abilities [9, 10]. In this study, we found that immortalized STEC line between passages 10 and 20 possessed a complete capacity for differentiation and the production of cytokines under Ali culture conditions. In practical application, the two systems can be used in concert, according to their advantages and disadvantages.

## Conclusions

Ali-cultured primary STEC and immortalized STEC systems were established in this study. The differentiation capacity and immunological function of cells in these two systems were systematically compared and analyzed for the first time. The two systems both possessed a complete capacity for differentiation and the production of cytokines in vitro, but they showed differences in cell morphology, tight junction formation and cilia differentiation. Ali-cultured immortalized STEC and primary STEC systems will be important tools for studying the interactions between hosts and respiratory pathogens, as well as for drug screening.

## Abbreviations

Ali: Air–liquid interface
BEGM: Bronchial epithelial growth media
BSA: Bovine serum albumin
DAPI: 2,4-Diamidino-2-phenylindole
HMDS: Hexamethyldisilazane solution
IFAs: Indirect immunofluorescence assays
IL-1β: IL-1beta
Mhp: *Mycoplasma hyopneumoniae*
NHBE cells: Normal human bronchial epithelial cells
NPTr: Newborn pig trachea
PBS: Phosphate-buffered saline
PRRSV: Porcine reproductive and respiratory syndrome virus
SEM: Scanning electron microscopy
SIV: Swine influenza virus
STEC: Swine tracheal epithelial cell
TLR: Toll-like receptor
TNFa: TNF alpha
USG: UltroserG serum substitute
ZO-1: *Zona occludens-1* protein
